# The Ozone-Iodine-Chlorate Clock Reaction

**DOI:** 10.1371/journal.pone.0083706

**Published:** 2013-12-26

**Authors:** Rafaela T. P. Sant'Anna, Emily V. Monteiro, Juliano R. T. Pereira, Roberto B. Faria

**Affiliations:** Instituto de Química, Universidade Federal do Rio de Janeiro, Rio de Janeiro, RJ, Brazil; University of Melbourne, Australia

## Abstract

This work presents a new clock reaction based on ozone, iodine, and chlorate that differs from the known chlorate-iodine clock reaction because it does not require UV light. The induction period for this new clock reaction depends inversely on the initial concentrations of ozone, chlorate, and perchloric acid but is independent of the initial iodine concentration. The proposed mechanism considers the reaction of ozone and iodide to form HOI, which is a key species for producing non-linear autocatalytic behavior. The novelty of this system lies in the presence of ozone, whose participation has never been observed in complex systems such as clock or oscillating reactions. Thus, the autocatalysis demonstrated in this new clock reaction should open the possibility for a new family of oscillating reactions.

## Introduction

A clock reaction is a special chemical phenomenon of which the Landolt clock reaction is among the best examples [1], [2]. Some of these reactions have captured the attention of a many students and others due to their sudden color changes that occur after a lag time or induction period. These clock reactions not only are beautiful but also belong to a class of nonlinear chemical phenomena including propagation of wave fronts, oscillating reactions and Turing structures, which are related to several natural processes and rhythms [3]–[6].

The definition of a clock reaction is a matter of dispute that remains unresolved. Some authors [7] consider the term ‘clock reaction’ appropriate only for reactions that present a stoichiometric ratio such that the end of the induction period is marked by the exhaustion of one reagent. However, the paradigmatic Landolt reaction between sulfite and iodate has been shown [8] to depend not only on the depletion of sulfite but also on the autocatalytic production of iodide and H^+^ to show the abrupt formation of iodine. Thus, we may consider any reaction to have clock-type behavior if it presents an induction period followed by an abrupt change in the concentration of some species. For simplicity, our use of the term ‘clock reaction’ indicates a reaction that exhibits clock-type behavior.

The chlorate-iodine clock reaction [10] is among the last clock reactions to be discovered (the last one is based on molybdenum blue [9]). This reaction presents a rapid consumption of iodine after an induction period that is inversely proportional to the initial chlorate and H^+^ concentrations but is independent of the initial iodine concentration. Galajda *et al.* [11] demonstrated that this reaction only occurs in the presence of the UV light from the deuterium lamp of a diode-array spectrophotometer, which breaks apart the iodine molecules, producing I• radicals that react with the chlorate.

In this work we show that addition of ozone to the chlorate-iodine system produces a clock reaction without requiring UV light.

## Materials and Methods

The reaction was followed at the iodine absorbance band maximum (λ = 460 nm, ε = 740 L mol^−1^ cm^−1^) [12] using a 10-mm optical path quartz cuvette and an Agilent 8453 UV-Vis spectrophotometer with only the tungsten lamp turned on (the deuterium lamp was turned off) to avoid exposing the sample to UV light. Some experiments were repeated following the reaction using a conventional double-beam Cary 1E spectrophotometer with identical results. Each experimental curve represents a set of several curves obtained for each set of initial reagent concentrations as indicated in the legends.

The ozone solutions were prepared by feeding high-purity oxygen into a commercial ozone generator. The ozone was bubbled in 0.6 mol L^−1^ perchloric acid to reduce the rate of ozone decomposition and obtain a concentration of approximately 3.0×10^−5^ mol L^−1^. The ozone concentration was measured using its absorbance at 260 nm (ε = 3000 L mol^−1^ cm^−1^) [13].

All reagents were used as received. The iodine was resublimed for some experiments, but the results were identical. All solutions were prepared using conductivity water (minimum 18 MΩ), and all experiments were conducted at 25±0.1°C.

A simulation of the proposed mechanism was developed via the numerical integration of the model presented in Table 1 using a semi-implicit Runge-Kutta method [14] codified in Turbo Pascal.

**Table 1:** Mechanism for the ozone-iodine-chlorate clock reaction.

Number^a^	Reaction	Rate law^b^
1*	I_2_ + H_2_O ↔ HOI + I^−^ + H^+^	1.98×10^−3^ [I_2_]/[H^+^] - 3.67×10^9^ [HOI][I^−^]
2*	I_2_ + H_2_O ↔ H_2_IO^+^ + I^−^	5.52×10^−^^2^ [I_2_] - 3.48×10^9^ [H_2_IO^+^][I^−^]
3*	HClO_2_ + I^−^ + H^+^ → HOI + HOCl	7.8 [HClO_2_][I^−^]
4**	HClO_2_ + HOI → HIO_2_ + HOCl	6.9×10^7^ [HClO_2_][HOI][H^+^]
5*	HClO_2_ + HIO_2_ → IO_3_^−^ + HOCl + H^+^	1.0×10^6^ [HClO_2_][HIO_2_]
6*	HOCl + I^−^ → HOI + Cl^−^	4.3×10^8^ [HOCl][I^−^]
7*	HOCl + HIO_2_ → IO_3_^−^ + Cl^−^ + 2 H^+^	1.5×10^3^ [HOCl][HIO_2_]
8*	HIO_2_ + I^−^ + H^+^ ↔ 2 HOI	1×10^9^ [HIO_2_][I^−^][H^+^] – 22 [HOI]^2^
9*	2 HIO_2_ → IO_3_^−^ + HOI + H+	25 [HIO_2_]^2^
10*	HIO_2_ + H_2_IO^+^ → IO_3_^−^ + I^−^ + 3 H^+^	110 [HIO_2_][H_2_IO^+^]
11*	HOCl + Cl^−^ + H^+^ ↔ Cl_2_ + H_2_O	2.2×10^4^ [Cl_2_][H^+^] - 22 [HOCl][Cl^−^]
12*	Cl_2_ + I_2_ +2 H_2_O → 2 HOI + 2 Cl^−^ + 2 H^+^	1.5×10^5^ [Cl_2_][I_2_]
13*	Cl_2_ + HOI + H_2_O → HIO_2_ + 2 Cl^−^ + 2 H^+^	1.0×10^6^ [Cl_2_][HOI]
14*	HClO_2_ ↔ ClO_2_^−^ + H^+^	2×10^8^ [HClO_2_] - 1×10^10^ [ClO_2_^−^][H^+^]
15*	HOI + H^+^ ↔ H_2_OI^+^	1×10^10^ [HOI][H^+^] - 3.4×10^8^ [H_2_IO^+^]
16*	I_2_ + I^−^ ↔ I_3_^−^	5.52×10^9^ [I_2_][I^−^] - 7.5×10^6^ [I_3_^−^]
17	O_3_ + I^−^ + H^+^ → HOI + O_2_	1.2×10^9^ [O_3_][I^−^][H^+^]
18	ClO_3_^−^ + HIO_2_ → IO_3_^−^ + HClO_2_	20 [ClO_3_^−^][HIO_2_][H^+^]
19	ClO_3_^−^ + H_2_IO^+^ + H^+^ → HIO_2_ + HClO_2_ + H^+^	1.4 [ClO_3_^−^][H_2_IO^+^][H^+^]

a) * Reactions taken from Lengyel *et al.* [16]. ** Modified from the Lengyel *et al.* [16] model by including the [H^+^] effect in the rate law.

b) Rate constant units are s^−1^, L mol^−1^ s^−1^, L^2^ mol^−2^ s^−1^ for the first-, second- and third-order processes, respectively.

## Results and Discussion

**Figure 1:**
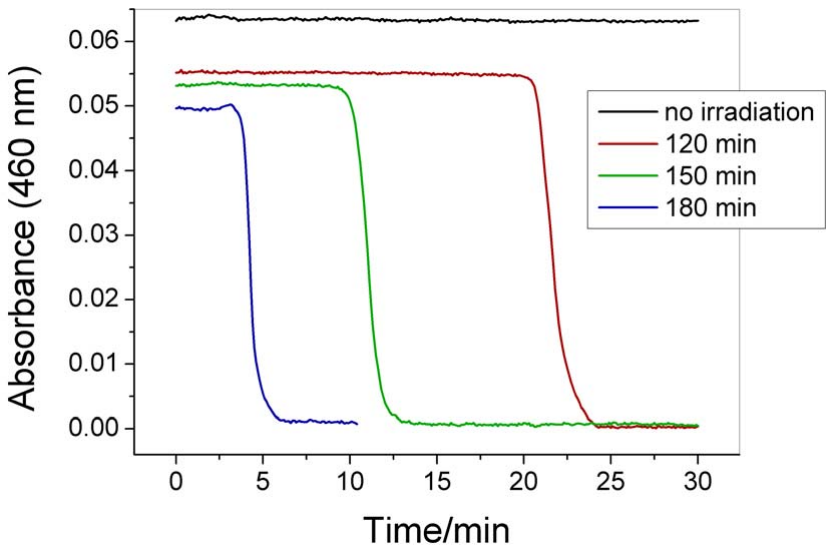
Effect of the iodine solution irradiation time on the clock time of the chlorate-iodine clock reaction. A 2.0×10^−4^ mol L^−1^ iodine solution was irradiated using the deuterium lamp of a diode array spectrophotometer during the indicated time. The reaction was followed at 460 nm using a conventional double-beam spectrophotometer. The initial concentrations after mixing the reactants in the cuvette were as follows: [NaClO_3_] = 0.0251 mol L^−1^, [HClO_4_] = 0.948 mol L^−1^, and [I_2_] = 7.3×10^−5^ mol L^−1^. The reactants were added to the solutions in the following order: NaClO_3_, irradiated iodine solution, water, and HClO_4_.

While repeating the Galajda *et al.* [11] experiments, we attempted to place a quartz cuvette containing an aqueous iodine solution into a diode array spectrophotometer to illuminate it with the UV light from the deuterium lamp. After a given irradiation time, we removed some of this solution from the diode array spectrophotometer and mixed it with the other reagent solutions in another cuvette inside a conventional double-beam spectrophotometer. Surprisingly, the system clocked. Additional experiments indicated that the observed clock time was inversely proportional to the time that the iodine solution was exposed to UV light as depicted in Figure 1.

Considering that the lifetime of the I• is small based on its re-combination rate (2 I• (aq) → I_2_ (aq); *k* = 8×10^9^ L mol^−1^ s^−1^ [15]), these radicals could not survive long enough (at least 10 s) to remove the cuvette containing the iodine solution from the diode array spectrophotometer, pipette this solution and mix it with the other solutions. In another experiment, we placed pure water into a quartz cuvette in the diode array spectrophotometer and irradiated it for a given time (90 min, 120 min or 150 min). Then, we mixed this irradiated water with the iodine, chlorate and perchloric acid solutions and observed that this system also clocked. The clock time was also inversely proportional to the irradiation time (figure not shown).

Because UV light is not expected to have any effect on water, one can naturally suppose that UV light split the dissolved oxygen (O_2_ + *hν* → 2 O) and formed ozone (O_2_ + O → O_3_) that could then participate in a sequence of reactions to produce the clock behavior. This interpretation was confirmed by preparing an ozone solution, mixing it with the other reagents and observing the clock behavior.

The addition of a hydrogen peroxide solution did not produce clock behavior, which suggests that we cannot assume any oxidizing species will produce this type of nonlinear behavior. The effect of adding HOCl or chlorite (which becomes HClO_2_) as observed by Galajda *et al.* [11] should be due to the participation of these species in the autocatalytic sequence of reactions as shown by the proposed mechanism (Table 1).

**Figure 2:**
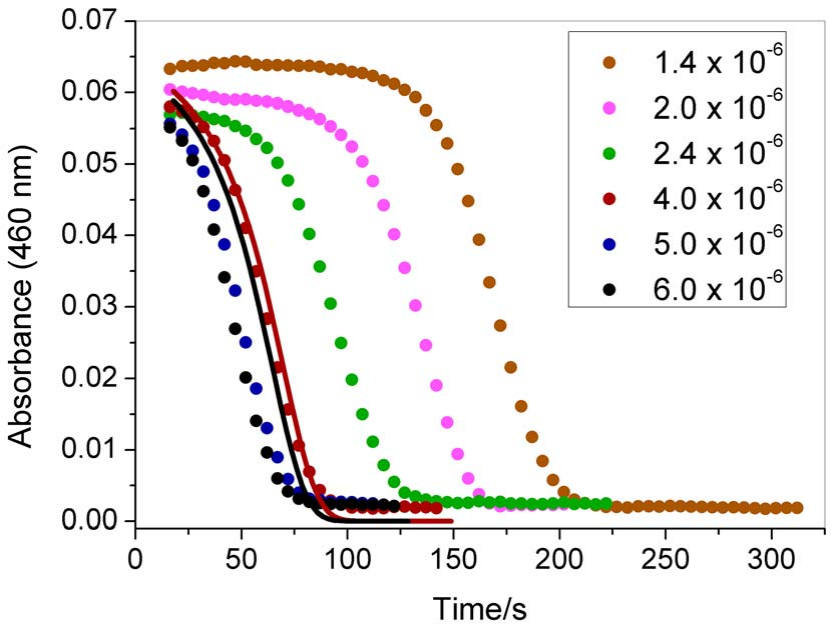
Effect of the ozone concentration on the clock time. The initial concentrations after mixing the reactants in the cuvette were as follows: [O_3_] as indicated in the figure (mol L^−1^), [NaClO_3_] = 0.0251 mol L^−1^, [HClO_4_] = 0.474 mol L^−1^, and [I_2_] = 8.8×10^−5^ mol L^−1^. Experimental data (symbols); modeling results (continuous line).

**Figure 3:**
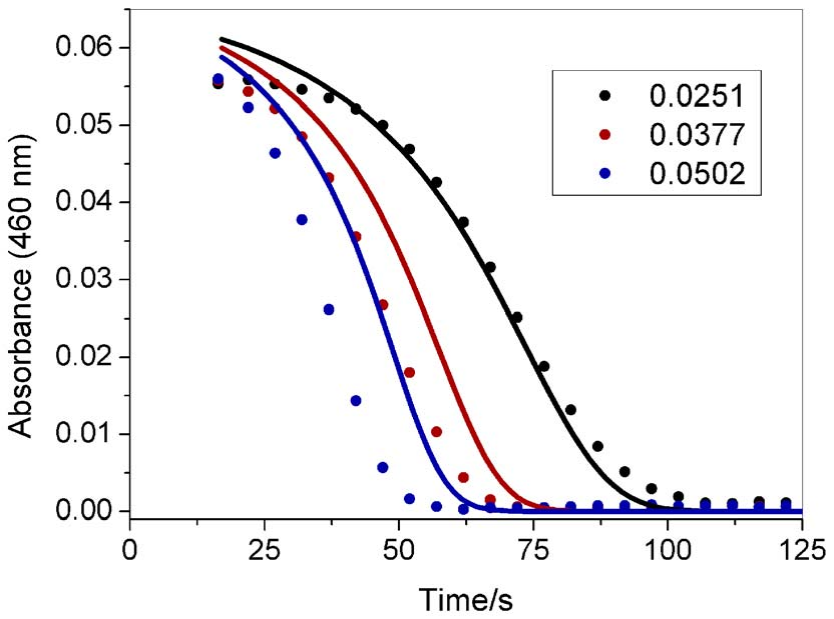
Effect of the chlorate concentration on the clock time. The initial concentrations after mixing the reactants in the cuvette were as follows: [NaClO_3_] as indicated in the figure (mol L^−1^), [O_3_] = 3.0×10^−6^ mol L^−1^, [HClO_4_] = 0,474 mol L^−1^, [I_2_] = 8.8×10^−5^ mol L^−1^. Experimental data (symbols); modeling results (continuous line).

**Figure 4:**
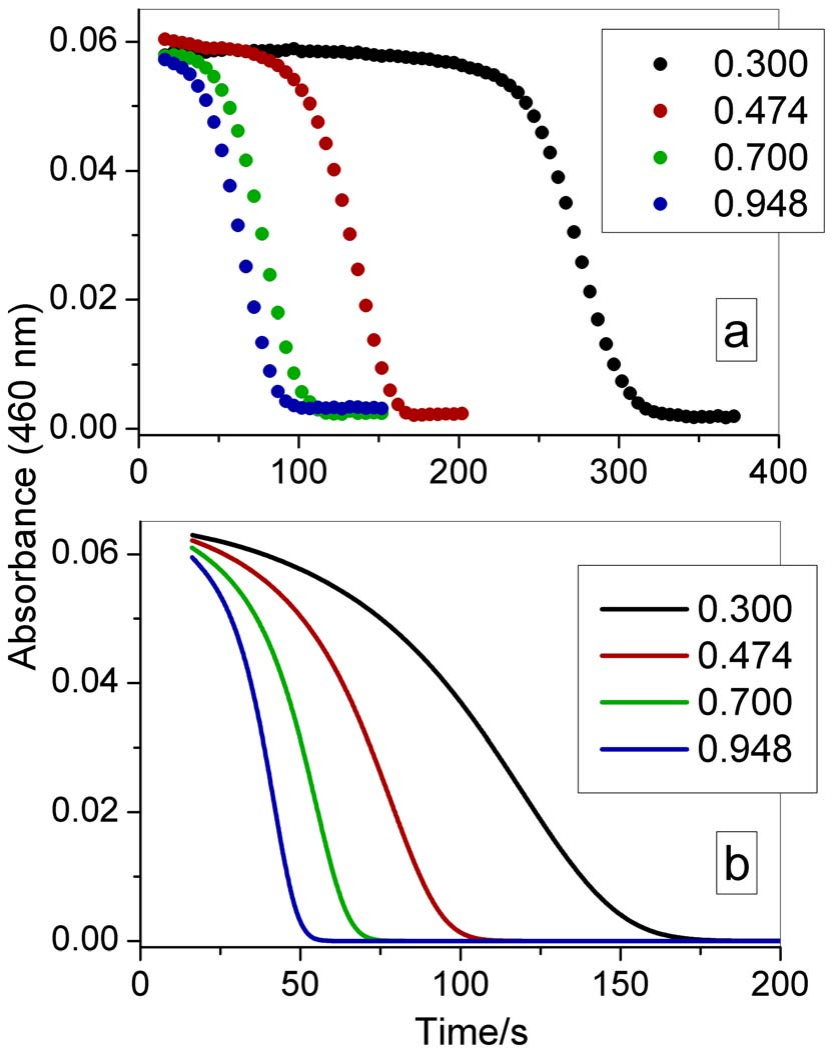
Effect of the acid concentration on the clock time. The initial concentrations after mixing the reactants in the cuvette were as follows: [HClO_4_] as indicated in the figures (mol L^−1^), [O_3_] = 2.0×10^−6^ mol L^−1^, [NaClO_3_] = 0.0251 mol L^−1^, [I_2_] = 8.8×10^−5^ mol L^−1^. (a) experimental data (symbols); (b) modeling results (continuous line).

**Figure 5:**
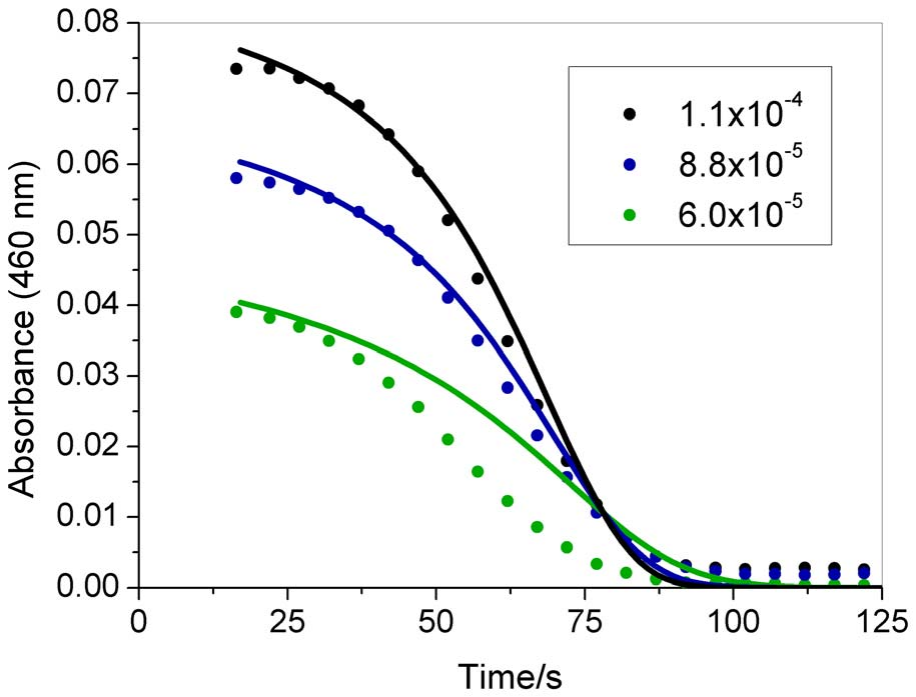
Effect of the iodine concentration on the clock time. Initial concentrations after mixing the reactants in the cuvette: [I_2_] as indicated in the figure (mol L^−1^), [NaClO_3_] = 0.0251 mol L^−1^, [HClO_4_] = 0.474 mol L^−1^, [O_3_] = 4.0×10^−6^ mol L^−1^. Experimental data (symbols); modeling results (continuous line).

The effect of the initial ozone, chlorate, acid, and iodine concentrations on the induction period is demonstrated in Figures 2 to 5. All curves begin at 15 s, which is the time required to mix the reagents in the cuvette and put it in the cuvette holder inside the spectrophotometer. The reagents were added using a digital pipette in the following order: water, NaClO_3_, iodine, HClO_4_ and ozone. As indicated in these figures, increasing the initial concentrations of these species reduces the induction period, except iodine which does not change the clock time but increases the initial absorbance.

The observed clock behavior can be understood by considering that ozone reacts with iodide to form HOI, which is a key species in producing the nonlinear autocatalytic behavior as previously demonstrated [16]. The full mechanism presented in Table 1 contains a core of reactions (Reactions 1 to 16) proposed by Lengyel *et al.* [16] between chlorine and iodine with species that provide the autocatalytic path necessary for the clock behavior. Although this mechanism core only qualitatively reproduces the chlorite-iodide clock behavior [17], it remains the best available model for this system. To this core group of reactions we added Reaction (17) between ozone and iodide [13], and added Reactions (18) and (19) to introduce the chlorate, as we made in our first model of the chlorate-iodine clock reaction [10], but with slightly different rate constants. To obtain qualitative agreement as demonstrated in Figure 4 for the acid concentration effect, we included one additional H^+^ in the rate laws for Reactions (4) and (19). In Reaction (4), this additional H^+^ accounts for the shifts in the [ClO_2_^−^]/[HClO_2_] and [HOI]/[H_2_OI^+^] ratios that should be affected by changes in [H^+^] as indicated by Jowsa *et al.* [17]. The same can be said for Reaction (19), which can be affected by the protonation of chlorate or some reaction intermediate. Other modifications of the manner in which H^+^ participates in other reactions could improve the agreement in the acid concentration effect (Figure 4), but we decided to minimize these modifications, following only the suggestions made by Jowsa *et al.* [17]. As indicated in Figures 2 to 5, the proposed mechanism can reproduce the behavior observed in the experiments, demonstrating good agreement with the chlorate and iodine effect. For the ozone effect (Figure 2), the model produces good agreement for [O_3_]_0_ = 4×10^−6^ mol L^−1^, but for other ozone concentrations, the agreement is only qualitative. For the acid concentration effect (Figure 4), the model produces a shorter clock time than the experimental value, but is still in good qualitative agreement.

Additional experiments have indicated that mixing ozone and iodine solutions decreased only the I_3_^−^ bands at 350 and 288 nm, indicating the oxidation of iodide (Reaction 17) followed by a shift in Reaction (16) to the left, allowing one to conclude that the ozone does not react directly with the iodine. Because the reaction between ozone and iodide has a high rate constant, we did not include the reactions between ozone and other low-concentration species in the model.

In conclusion, without UV light, the addition of ozone to the chlorate-iodine system initiates an autocatalytic sequence of reactions that results in the rapid consumption of iodine after an induction period. However, if UV light is present, its effect over the iodine molecules cannot be ignored, especially in systems containing oxychlorine species, as observed in the photoinduced reactions between ClO_2_• and I_2_ [18] and between ClO_3_^−^ and I_2_ [10], [11].

To our knowledge, this is the first time that the participation of ozone in a system to produce complex nonlinear behavior has been demonstrated. This research can serve as the first step to discover oscillating reactions or other nonlinear dynamic behavior involving ozone.
